# Case report: Microsurgical replantation of heel skin and subcutaneous tissue

**DOI:** 10.3389/fsurg.2022.815125

**Published:** 2023-01-06

**Authors:** Jian Zhou, Shusen Chang, Chengliang Deng, Guangtao Huang, Wenhu Jin, Wei Chen, Zairong Wei

**Affiliations:** Department of Plastic Surgery, Affiliated Hospital of Zunyi Medical University, Zunyi, China

**Keywords:** microsurgical, replantation, tissue avulsion, composite tissue, reconstruction

## Abstract

A 36-year-old healthy male patient was presented to the emergency room 3 h after experiencing a laceration to the left foot caused by a porcelain shard. The defect measured 7.5 × 6.0 × 0.8 cm, and the composite amputated tissue consisted of skin and subcutaneous layers. The terminal branch of the lateral calcaneal artery was first anastomosed end-to-end to the corresponding artery in the wound defect. The lateral calcaneal nerve was anastomosed after blood flow was restored. The two lateral veins were anastomosed end-to-end to the corresponding veins in the wound defect. Postoperatively, 1.0 × 1.5 cm area of skin necrosis was present at the distal end of the tissue, which healed smoothly after two weeks of dressing changes. The patient had retained excellent aesthetic and functionality by the 37 month follow up. Although such isolated amputation is relatively rare, microsurgical replantation is, thus, a feasible option in the management of heel amputation.

## Introduction

Tissue avulsion caused by trauma is difficult to manage, with various methods of repair available. In principle, microsurgical techniques for tissue replantation using microsurgical techniques are the best option, but the degree of difficulty of replantation differs among regions. Clinically mature techniques are available for replantation of severed fingers, scalp avulsion, severed ears, or a severed penis (1–4). Due to the relatively consistent anatomy of the blood vessels and nerves, clinical reports are much more common. However, few cases of heel replantation have been reported, and none have described replantation of heel skin and subcutaneous tissue amputation (5–7). The present report describes the case of a patient who underwent replantation with amputated skin and subcutaneous tissue.

## Case report

**Figure 1:**
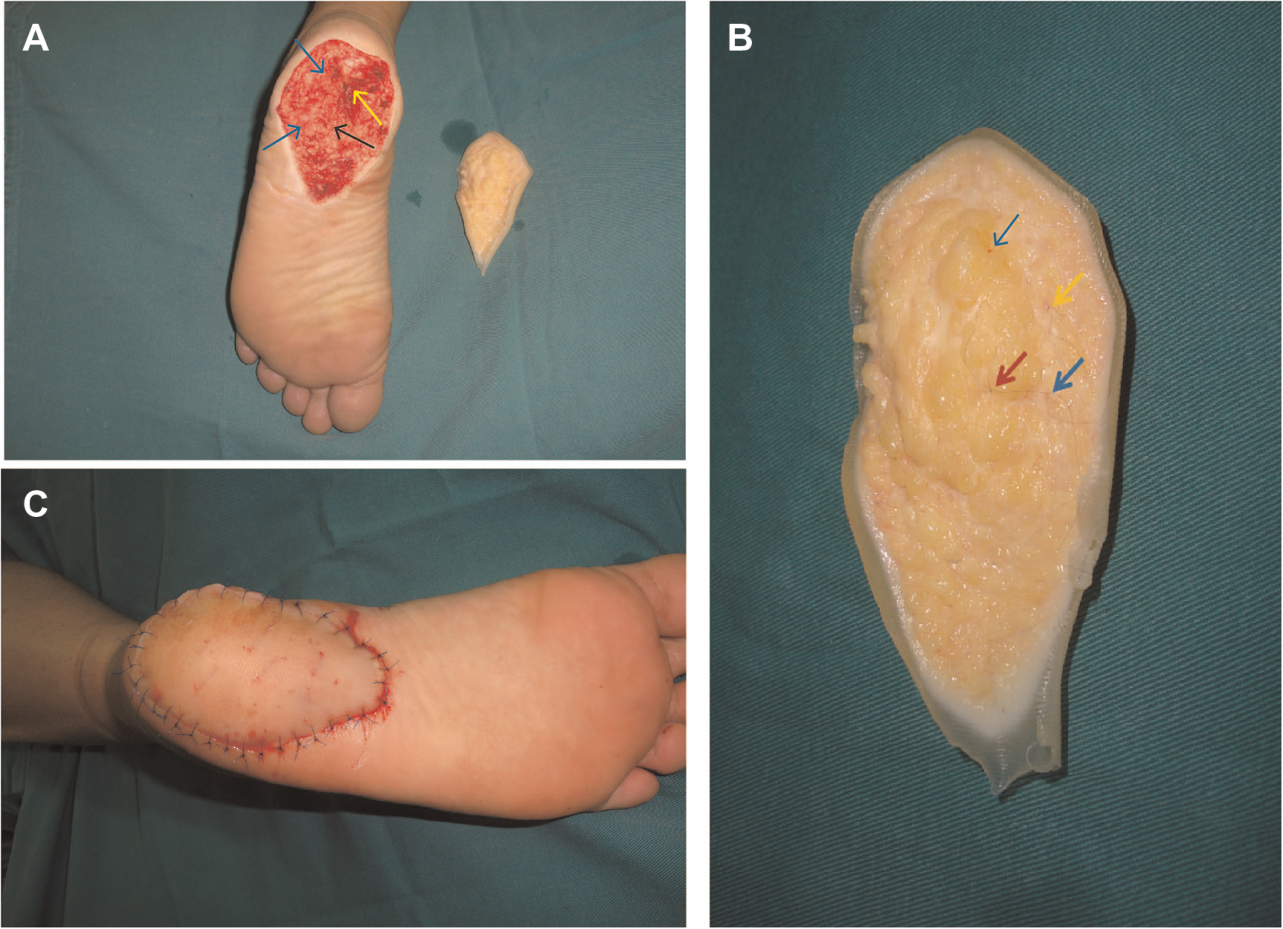
A 36-year-old male with a laceration to the left foot injury from broken porcelain shard. (**A**) Heel skin defect wound (7.5 × 6.0 × 0.8 cm), no bones and tendons exposed (Black arrows indicate arteries, yellow arrows indicate nerves, and blue arrows indicate veins). (**B**) The blood vessels and nerves are marked with 11-0 nylon under the microscope. (Red arrows indicate arteries, yellow arrows indicate nerves, and blue arrows indicate veins). (**C**) The replanted tissue immediately appeared healthy, following the operation.

A 36-year-old healthy male patient was presented to the emergency room 3 h after experiencing a laceration to the left foot caused by a porcelain shard. The patient accidentally stepped on a porcelain bedpan while washing his feet, resulting in a wound bed measuring 7.5 × 6.0 × 0.8 cm, with no bone or tendon exposure (Figure 1A). The patient has no history of smoking, drinking, taking medication, and no history of diabetes or vascular related diseases. The subject provided written informed consent for this case report.

Normal saline was used to rinse the tissue pieces 4–5 times. About 2 mm of tissue at the wound edge was removed, and 0.05% iodophor was used to wash the tissue block 2–3 times. The amputated tissue was clean and in good condition. The vessels and nerves were identified and marked under a microscope on a back table while the patient was prepared for operation. Artery, vein, and nerve repair was identified and marked (Figure 1B).

A pneumatic tourniquet was applied to the left thigh but not inflated. The patient was placed in prone position and ankle plantarflexion was fixed at 30°. The terminal branch of the lateral calcaneal artery was dissected and marked under a microscope with the pulsatile bleeding point as the target point. The artery was centrally located in the wound and was approximately 0.4 mm in diameter; the nerve was then identified near the artery and marked. The peripheral veins (diameter, 0.6 mm) were easier to approach from the medial or lateral margin of the heel wound (Figure 1A).

The amputated tissue was placed in the wound, and the medial and distal edges of the amputated tissue were secured with temporary sutures to prevent avulsion. The anastomosis of blood vessels and nerves was completed under a microscope with a magnification of 12 times. The tourniquet was inflated to stop bleeding, then the terminal branch of the lateral calcaneal artery was pulled out of the tissue and infibulated with a microvascular clamp. The artery was first anastomosed end-to-end to the corresponding artery in the wound defect using 11-0 nylon sutures. Next, the tourniquet was released and the anastomotic artery was observed to confirm whether the blood flow was restored. The lateral calcaneal nerve was anastomosed after blood flow was restored. Then, the artery was infibulated with a microvascular clamp and the two lateral veins were anastomosed end-to-end to the corresponding veins in the wound defect using 11-0 nylon sutures. With the anastomosis complete, all temporary sutures were removed and the skin margin was sutured carefully with 5-0 Prolene (Figure 1C), ensuring no disruption of the arterial and venous anastomoses.

**Figure 2:**
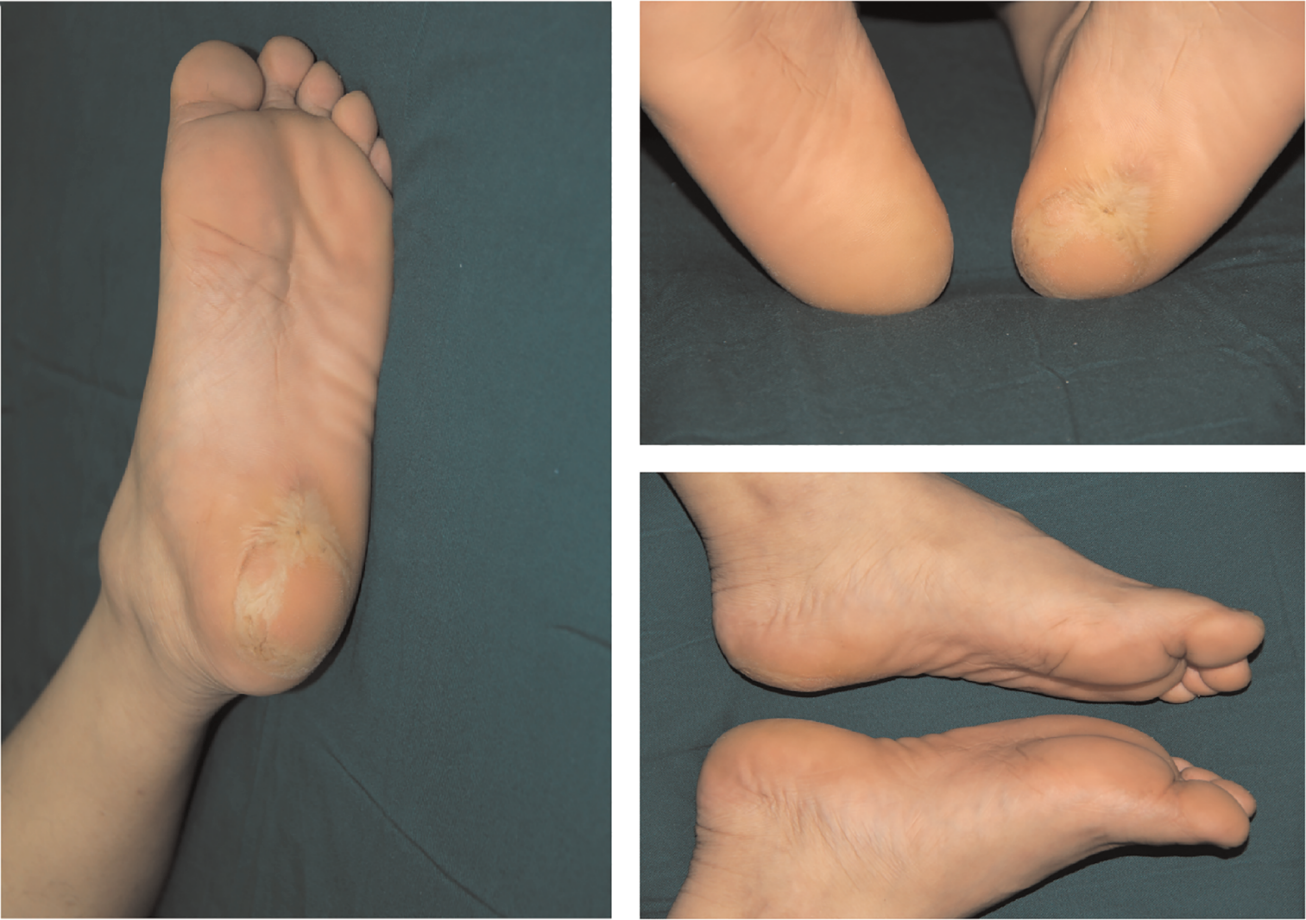
The patient demonstrated excellent aesthetic and functionality 37 months following the operation.

Postoperative treatment included intravenous (IV) cefamandole nafate (2 g every 8 h for 3 days), heparin (continuous IV drip of 125,000 IU/day for 7 days), low-molecular-weight dextran (continuous IV drip of 1,000 ml/day for 3 days), and IV papaverine hydrochloride (30 mg every 8 h for 7 days). An Armour oven lamp was used to maintain warmth (40 W at a distance of 40 cm for 7 days). The stitches were removed 14 days after surgery. A 1.0 × 1.5 cm area of skin necrosis present after surgery at the distal end of the tissue healed smoothly after 2 weeks of dressing changes. Rehabilitation training for left foot function was performed 45 days after surgery. Follow-up examination at 37 months showed excellent aesthetic and functional results, with no development of trophic ulceration (Figure 2). The static 2-point discrimination results were similar between the replanted heel (16.0–20.0 mm) and the contralateral side (10.0 mm). Most importantly, the patient can wear shoes and walk without discomfort.

## Discussion

Through this study, it was found that microsurgical replantation is a possible means of managing heel amputation, and the benefits of this method outweigh the challenges. Managing heel amputation is critical because of the essential role the heel has in the functioning of the foot. Much adipose tissue present in the subcutaneous tissue is separated by fiber bundles that are elastic and can fully absorb and resist impact and vibration from external forces. When there is a soft tissue defect in the heel caused by an accident, or other means, repair requires restoration of a tough, wear-resistant, and non-bulky textured tissue with good sensation. If the soft tissue condition is good and the patient prefers the defect to be repaired, the surgeon should make every effort to replant the tissue, as this is the best choice for repair and reconstruction.

Young healthy patients with traumatic superficial injuries usually heal without surgery. However, when the defect area is large, non-surgical treatment takes a long time and scars formation, which seriously affects the work and life of the patient, and the function of the affected limb was discounted. The wound bed in the present case was large and the patient was unwilling to receive non-surgical treatment. Two alternatives to replantation in the present case could have included conversion of the amputated tissue into a skin graft and skin flap repair (8, 9). The amputated portion could be revised, trimmed of subcutaneous fat, and reapplied to the wound bed as a full-thickness skin graft. Because of the thickness of keratin in the heel, the probability of survival is greatly reduced, and survival could result in a local depression. The skin graft may not be able to withstand the pressure, friction, and shear forces from footwear that the heel pad experiences; in this case, ulcerations and other adverse outcomes become more likely (10). In conjunction with the requirements described here, the medial plantar flap is the first choice for reconstruction, but inevitably causes another injury to the affected foot, and produces a flap donor site that may require a skin graft (11, 12). Sometimes cutting the flap will destroy the plantar aponeurosis, with a slight effect on foot function but increased morbidity at the donor site. Microsurgical replantation can fulfill the purpose of repair in one operation and avoid sacrificing other donor sites. It is critical to restore original function and replantation is the optimal repair method.

The injury in the present patient was caused by a porcelain shard; the tissue mass remained intact, with no injury to the bone or tendon, and there was no exposure. For successful replantation, it is particularly important to search for blood vessels. The blood supply in the heel is formed by the posterior tibial artery, peroneal artery, and terminal branches of the medial and lateral calcaneal arteries (13). The vessel caliber is suitable for anastomosis, and the generous venous and nerve distribution ensure that replantation is feasible.

The success of the operation depends on its details and what the procedure entails. As many arteries and veins as can be identified should be anastomosed. One artery and two veins were anastomosed in this case because the blood supply in the amputated tissue was found to be adequate immediately after surgery. Effective fixation is also essential to ensure success. When anastomosing the lateral calcaneal artery and nerve, the medial margin and distal end of the amputated tissue should be fixed on the wound. To prevent vascular and nerve avulsion caused by pulling during vein anastomosis, the medial side of the anastomosed lateral calcaneal artery and nerve should also be fixed. The cause of distal necrosis in this case was a severe tissue contusion and insufficient arterial blood supply, but these occurrences had no significant effect on the overall appearance and function during long-term follow-up.

Although our results were encouraging, several disadvantages needed to be considered. Firstly, this method requires ultramicro technology, and therefore, it is essential that the surgeon is experienced. In addition, in the present case, this patient is young and healthy, without high-risk factors affecting tissue survival, which is also a key factor for the success of the operation. However, for patients with high-risk factors affecting tissue survival, such as the elderly, diabetes, peripheral vascular disease, etc., whether this microsurgical replantation method is feasible still requires further research. Moreover, not all isolated tissue blocks are suitable for microscopic replantation. It is difficult to dissect anastomosing blood vessels in thin tissue with small amount of subcutaneous adipose tissue, so microscopic replantation cannot be performed. For patients with nerve anastomosis, more objective testing methods should be used to evaluate the recovery of neurological function, not just two-point discrimination.

In conclusion, the present case demonstrates that microsurgical replantation is a feasible option in the management of heel amputation. The technical difficulties were offset by the positive results. Although such isolated amputation is relatively rare, this study shows that replantation can be achieved with microsurgery.

## Data Availability

The original contributions presented in the study are included in the article/Supplementary Material, further inquiries can be directed to the corresponding author/s.
